# The Comparison of Color Stability of Aloe Vera Gel and Chlorhexidine Solution on Acrylic Teeth

**DOI:** 10.1155/2022/6196803

**Published:** 2022-10-21

**Authors:** Farhang Mahboub, Amin Nourizadeh, Armin Izadpanah

**Affiliations:** ^1^Department of Prosthodontics, Faculty of Dentistry, Tabriz University of Medical Sciences, Tabriz, Iran; ^2^Faculty of Dentistry, Tabriz University of Medical Sciences, Tabriz, Iran

## Abstract

**Background:**

There is insufficient knowledge about aloe vera color change property on acrylic teeth compared to other denture cleaners, especially chlorhexidine solution (CHX).

**Objective:**

The present study aimed to compare the color change property of the aloe vera and chlorhexidine solution on denture acrylic teeth.

**Methods:**

This study was experimental in vitro study design conducted in the dental laboratory of Tabriz University of Medical Sciences, Iran, 2021. The sample size in each group was 21 acrylic teeth which were randomly divided into aloe vera gel 100% and chlorhexidine solution 2% groups. Tooth colors were measured by a spectrophotometer (Spectro Shade Micro, MHT S.P.A., Milan, Italy). The immersion time in both groups was 36 hours. Data analyzed using an independent *t*-test was used at a 95% of confidence level.

**Results:**

The comparison of the total color differences (Δ*E*) between the two groups was not statistically significant after 36 hours of immersion (*P*=0.440). In the chlorhexidine group, the brightness of teeth was lower than that in the aloe vera group (*P*=0.002).

**Conclusions:**

Clinicians should be aware that aloe vera gel does not change the color of denture acrylic teeth after 36 hours of immersion similar to the chlorhexidine solution. For patients who cannot use chlorhexidine, aloe vera gel is a good cleaner for denture acrylic teeth without worrying about the color change of the denture. It can be considered an acceptable cleaner for denture acrylic teeth.

## 1. Introduction

Dentures are periodically exposed to many bacteria, viruses, and fungi in the human mouth. Potential contamination of dentures with various pathogens in the laboratory is another source of contamination of dentures. The reuse of contaminated dentures due to the surface roughness of the resin and the penetration of microorganisms inside acts as a source of gingival infection [1, 2]. Therefore, it seems necessary to use disinfectants to clean the denture surface. Different denture cleaners have been introduced with different effects, which are mainly divided into two main categories, chemical and mechanical methods [3, 4].

The chemical method consists of solutions with antibacterial and antifungal actions that can be used alone or with the mechanical method or ultrasonic method [5]. Daily use of some denture cleansers can affect the physical and mechanical properties of denture base material as well as change their color. Teeth color change is a big esthetic problem and changing that may cause dissatisfaction in the patient [6, 7].

Color stability is the main property of many dental materials which can be measured by the color perceived from the object or colorimetry based on the digital technique. The standard Commission International de l'Eclairage (CIE *L*^*∗*^*a*^*∗*^*b*^*∗*^) color system as a digital technique has been recommended by the American Dental Association (ADA). According to this system, all colors in nature are obtained through the blending of 3 basic colors, namely, red, blue, and green, in certain proportions. This technique is widely used by researchers in dentistry to study the color of dental materials [8].

Sodium hypochlorite, chlorhexidine, and glutaraldehyde are commonly used as disinfectants. These solutions have several unpleasant side effects, such as cytotoxic activity on human cells, burning sensation in the mouth and throat, eye and skin irritation, denture discoloration, environmental pollution, and side effects on health workers [6, 9, 10].

Therefore, finding a natural and environmentally friendly alternative material with a high disinfectant effect comparable to routine disinfectants is a necessity [7]. In this regard, the use of complementary medicine is one of the appropriate options that are more and more considered by researchers in the field of dentistry [11]. One of the most important cleaners, which has antibacterial, antifungal, and antiviral properties, is the aloe vera plant. [12,13] Furthermore, using aloe vera does not change the shear strength of the composite resin [14,15].

Aloe vera is a natural substance that has been used for medical purposes at different ages and can also be used in the field of dentistry as a disinfectant with no or minimal side effects. It is also easily accessible, less expensive, and most importantly, 100% biodegradable, and does not harm the environment [16].

Chlorhexidine (CHX) is a solution with high antibacterial properties and can be used to clean dentures [17, 18]. Chlorhexidine can be used in both soluble and gel forms. Due to the lower fluidity of chlorhexidine gel, it is mostly used for root canal preparation, treatment of periodontal diseases and surgeries, and as a solution due to its higher fluidity, and in terms of cost-effectiveness, it is used for cleaning dentures or mouthwashes but in the long-term use, it causes tooth color change [19, 20]. Therefore, there is a need to find a cleaner that has less color change.

There is insufficient knowledge about aloe vera color change property on acrylic teeth compared to other denture cleaners, Therefore, this study aimed to compare the color change property of aloe vera gel and chlorhexidine solution on denture acrylic teeth.

## 2. Materials and Methods

This study was experimental in vitro study design conducted in the dental laboratory of Tabriz University of Medical Sciences, Iran, 2021.

This study aimed to compare the effect of aloe vera gel 100% with chlorhexidine 2% on one primary endpoint color change of acrylic denture teeth (the Δ*E* value) after 36 hours of immersion. Under controlled settings, the minimum difference in color tooth perceived by human viewers is a Δ*E* value of one [21]. In the present study, the acceptable level of 1 < Δ*E* ≤ 2. *P* value equal and more than 0.05 is the probability that the null hypothesis is true.

We do not access a similar study showing the effect of aloe vera gel 100% and chlorhexidine 2% on the color change of acrylic denture teeth. Therefore, the sample size was calculated based on the antimicrobial effect of aloe vera gel 100% which was reported in the study by Goud et al. [18].

Considering the 95% confidence level (*Z*_1_ _−_ _*α*_ = 1.96), the first type error *α* = 0.05, the test power of 80% (Z_1_ _−_ _*β*_ = 0.80), and the antimicrobial variable with the mean and standard deviation in the aloe vera gel (*μ*_2_ = 2.88 and *S*_2_ = 0.09) and % sodium hypochlorite (*μ*_1_ = 2.76 and *S*_1_ = 0.18) and using the formula for calculating the sample size in two independent groups, the total sample size was calculated to be 21 teeth for each group.

(1) $$\begin{array}{c} Z_{\left(1-\alpha /2\right)}=1.96\text{,}\\ Z_{\left(1-\beta \right)}=0.80\text{,}\\ S_{1}^{2}={\left(0.18\right)}^{2}\text{,}\\ S_{2}^{2}={\left(0.09\right)}^{2}\text{,}\\ {\left(\mu_{1}-\mu_{2}\right)}^{2}={\left(2.88-2.76\right)}^{2}\text{,}\\ N=\frac{{\left(Z_{\left(1-\alpha /2\right)}+Z_{\left(1-\beta \right)}\right)}^{2}\left(S_{1}^{2}+S_{2}\right)}{{\left(\mu_{1}-\mu_{2}\right)}^{2}}=21\text{.} \end{array}$$

Forty-two lower central or lateral acrylic teeth A2 (Iran, Tehran, Beta dent, and Novinvit) were prepared and randomly divided into two groups (aloe vera gel and chlorhexidine solution). The color experimenter did not know the names of the groups, and the samples were delivered under the names of A and B for spectrophotometric examination.

Before immersing the teeth in aloe vera gel and chlorhexidine solution, the baseline of the color of the samples was measured using a spectrophotometer (Spectro Shade Micro, MHT S.P.A., Milan, Italy); then, the first group was exposed to chlorhexidine solution 2% (Tehran, Iran, Marvaben) for 36 hours [18, 22], and the second group was exposed to aloe vera gel 100% in the same manner.

The chlorhexidine solution 2% has been prepared by Marvaben Company, and aloe vera gel 100% is a product of Barij Essence company, Tehran, Iran, respectively. The acrylic teeth are a product of Beta dent, and NovoInvent company, Tehran, Iran. The color change was measured with a spectrophotometer (Spectro Shade Micro, MHT S.P.A., Milan, Italy). Aloe vera gel has been produced by Tehran, Iran, Barij Essence company, and it is stored in a sealed bottle at 4°C. Each time, a certain amount of gel was poured into a clean container and the teeth were immersed in the gel.

After the end immersing time, the samples were rinsed with distilled water for 3 minutes and then dried, and immediately the amount of color change was measured by spectrophotometry so that the samples did not become dehydrated [22]. For standard calibration and to eliminate the effects of background color differences during the color measurement, black color was used as the background for each sample. The spectrophotometer (Spectro Shade Micro, MHT S.P.A., Milan, Italy) was used to measure the CIE *L∗a∗b∗*color parameters of the teeth. The initial color of each tooth was measured before immersion in the solutions. In the CIE *L∗a∗b* color space, the brightness is indicated by *L∗*, red-green by *a∗*, and blue-yellow by *b∗*). The device was calibrated before measurement, and the measurements were made three times for each tooth. The mean values were considered the final value. Color difference (CIELab) was calculated using the following formula:

(2) $$\begin{array}{c} \Delta E={\left[{\left(\Delta a\right)}^{2}+{\left(\Delta b\right)}^{2}+{\left(\Delta L\right)}^{2}\right]}^{1/2}. \end{array}$$

The Kolmogorov–Smirnov test was used to assess the normality of data distribution, and as a result of the test, our data had a normal distribution. Therefore, an independent *t*-test was used to compare the ΔE value between the two groups (chlorhexidine solution and aloe vera gel). The chi-squared test was used to compare the frequency distribution of teeth between the two groups. The analysis was performed at a 95% confidence level using SPSS 16.0 software (SPSS, Inc., Chicago, IL).

## 3. Results

**Figure 1:**
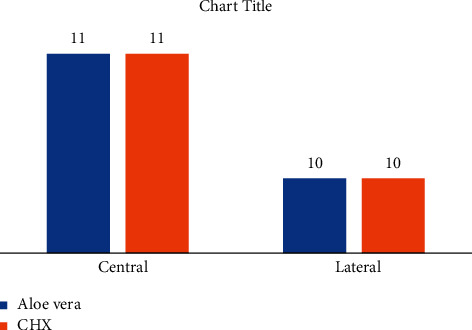
Frequency distribution of teeth in terms of two groups (aloe vera and CHX).

The results of Figure 1 showed that eleven central teeth and ten lateral teeth were distributed in each group (Figure 1).

Based on the chi-squared test, the frequency distribution of teeth was not statistically significant between the groups (Table 1).

**Figure 2:**
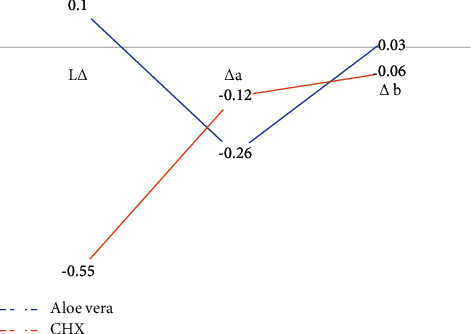
The comparison of Δ*L*, Δ*a*, and Δ*b* mean color difference.

The results of Figure 2 showed that the mean Δ*L* value in aloe vera and chlorhexidine solution groups were 0.10 and−0.55, respectively. In the chlorhexidine group, the brightness of teeth was lower than that in the aloe vera group. The mean Δ*a* value in the aloe vera and chlorhexidine groups were −0.26 and−0.12, respectively, which indicates that in the aloe vera group, we have slight changes in tooth color towards green. The mean Δ*b* value was 0.03 in the aloe vera group and −0.06 in the chlorhexidine group. The values of Δ*b* in the chlorhexidine group were negative and indicated slight changes in tooth color towards yellowness (Figure 2).

The mean ΔE value in aloe vera and chlorhexidine groups were 0.95 and 1.09, respectively. Based on an independent *t*-test, the comparison of the total color differences (Δ*E*) between the two groups was not statistically significant after 36 hours of immersion (*P*=0.440) (Table 2).

## 4. Discussion

This study aimed to compare the effect of the CHX 2% solution and aloe vera gel on the color stability of acrylic denture teeth. The results showed that after 36 hours of immersion, the mean of Δ*E* in the CHX group was not statistically significant compared to the aloe vera group, but the brightness change of teeth was significant.

The use of aloe vera as a complementary drug has a long history in dentistry. Based on the available evidence, the effective role of aloe vera in reducing gingivitis, [23] healing or preventing traumatic oral ulceration related to fixed orthodontics, [24] stability of dentin shear bond strength [25], and dental canal disinfection [26] is comparable with CHX solution and can be considered an alternative option to the CHX solution. Aloe vera is also more effective in disinfecting and retaining dentures compared to other traditional herbs, and no major side effects have been reported [27].

However, to the best of our knowledge, this is the first study to compare the properties of aloe vera gel on the color change of denture acrylic teeth with the CHX solution. Daily disinfection of dentures with chlorhexidine may not cause very serious side effects on the teeth in short term, but in long-term use, denture color change has been reported [28]. Suha Fahdil 2006 showed that immersion of pink acrylic resin in chlorhexidine solution for 6 continuous days has significant color changes compared to distilled water and saliva [29].

In the present study, the brightness of the teeth after immersion in chlorhexidine was significantly changed, which is consistent with the results of Suha Fahdil's study. According to the literature, the effects of tooth color change due to contact with aloe vera solution or gel are very limited. In this regard, Mahmiyah et al. showed that the whitening effect of saponin extracted from aloe vera on teeth color compared with oxygen peroxide 1% was significant. Saponins are high molecular weight glycosides that have a sugar arm attached to triterpene or steroidal aglycone [30].

Many saponins have cleaning properties and produce a stable foam in the water. In Mahmiyah's study, they prepared a 100% solution by dissolving 10 mg of saponin powder extracted from the aloe vera plant in 10 ml of distilled water. Then, saponin at a concentration of 50%, 25%, 12.5%, 6.25%, 3.125%, and 1.56% was diluted. 72 nondecayed caries teeth that have never been exposed to bleach immersed in each of these concentrations for 30 minutes, 45 minutes, and 60 minutes, then rinse with distilled water and dry with a tissue. Nine teeth were also exposed to 0.1% hydrogen peroxide solution at the same immersion time. The results showed that the most effective dose for teeth whitening was 100% saponin with an immersion time of 60 minutes, which increased the whitening by 2.56 times on average. The whitening effect of immersing the teeth in a solution of 0.1% H_2_O_2_ for 30 minutes was equal to immersing in a solution of 50% saponin for 30 minutes or 25% saponin for 45 minutes or 6.25% saponin for 60 minutes [30]. In the present study, the color change of the artificial teeth before and after immersion with aloe vera was small. Therefore, it did not match the results of the above study.

Incompatibility with the results can be due to differences in the type of teeth tested and differences in the aloe vera drug forms. In the present study, natural aloe vera gel was used, but in the above study, saponin extract extracted from aloe vera was tested.

Aloe vera contains disinfectants that have an inhibitory effect on fungi, bacteria, and viruses and prevents denture stomatitis. Saponins act as antimicrobials against bacteria, viruses, and fungi. Applying aloe vera gel twice a day for a few minutes on dentures and then rinsing with a solution of vinegar and water can loosen dental plaque and remove stains [31]. There are many uses for aloe vera in dentistry, and the use of aloe vera as a teeth cleaner is not harmful to swallow, and due to its reasonable price, many people with less access to oral health services can use it. It could be interesting in the future to test aloe vera in combination with other recently introduced compounds, such as paraprobiotics, lysates, and postbiotics to understand the better the usage of natural products [32–34].

The role of alternative medicine in oral health is discussed worldwide [11]. Although the positive effect of some alternative medicine is still an ongoing process, dental specialists can use these treatments in their practice along with other formal procedures as an integrative treatment approach to achieve better outcomes.

## 5. Limitations

The limitations of the present study included an in-vitro laboratory study used for testing the color stability of aloe vera gel and chlorhexidine solution on acrylic teeth, color change properties of aloe vera gel have only been tested on a single dose (100%), and the final limitation was the immersion time, which was 36 hours. It is suggested that the generalizability of the results take into account the limitations.

## 6. Implications for Future Research

It is suggested that a randomized clinical trial study be performed with different doses of aloe vera gel only or a combination of other complementary drug in different timings of immersion.

## 7. Conclusion

Clinicians should be aware that aloe vera gel does not change the color of denture acrylic teeth after 36 hours of immersion similar to the chlorhexidine solution. For patients who cannot use chlorhexidine, aloe vera gel is a good cleaner for denture acrylic teeth without worrying about the color change of the denture. It can be considered an acceptable cleaner for denture acrylic teeth.

## Figures and Tables

**Table 1:** The comparison of the frequency distribution of teeth between the two groups (aloe vera and CHX)

Variable			Group		*P* value^*∗∗*^
			Aloe vera	CHX^*∗*^	
Teeth	Central	Frequency	11	11	1
		Percent	50%	50%	
	Lateral	Frequency	10	10	
		Percent	50%	50%	

^*∗*^Chlorhexidine solution (CHX) and ^*∗∗*^chi-squared test.

**Table 2:** The comparison of mean Δ*E* color difference between the groups (independent sample test).

axis	Group	Sample size	Mean	SD^*∗*^	MD^*∗∗*^	CI 95%^*∗∗∗*^	*P* value
Δ*L*	Aloe vera	21	0.1	0.498	0.65238	0.25292, 1.05184	0.002
	CHX	21	−0.5524	0.75141			
Δ*a*	Aloe vera	21	−0.2667	0.20083	−0.14286	−0.31607, 0.03035	0.103
	CHX	21	−0.1238	0.3375			
Δ*b*	Aloe vera	21	0.0381	0.90137	0.10476	−0.43156, 0.64109	0.695
	CHX	21	−0.0667	0.81629			
ΔE^*∗∗∗∗*^	Aloe vera	21	0.9555	0.47837	−0.1383	−0.49725, 0.22065	0.44
	CHX	21	1.0938	0.65554			

^*∗*^Standard deviation, ^*∗∗*^mean difference, ^*∗∗∗*^confidence interval, and ^*∗∗∗∗*^Δ*E* color difference.

## Data Availability

The datasets used and analyzed during the current study are available from the corresponding author on reasonable request.
